# Understanding the Adsorption Behavior of Different Crystal Surfaces of Manganese Monoxide to Strontium Nitrate Solutions: A Molecular Dynamics Simulation

**DOI:** 10.3390/ma18081752

**Published:** 2025-04-11

**Authors:** Qingwei Xiang, Xingyu Yu, Kuixiang Guo, Chufeng Cheng, Xixiang Yue, Jingsong Wang, Yaochi Liu

**Affiliations:** 1 School of Civil Engineering, University of South China, Hengyang 421001, China; xqw18007383588@163.com (Q.X.); 18671965380@163.com (C.C.); sundori777@163.com (X.Y.); 2 School of Resources, Environment and Safety Engineering, University of South China, Hengyang 421001, China; yuxingyu1996@126.com; 3 Shanghai Urban Construction Design Research Institute (Group) Co., Ltd., Shanghai 200125, China; guokuixiang@sucdri.com; 4 College of Chemistry and Chemical Engineering, Central South University, Changsha 410083, China; liuyaochi72@163.com

**Keywords:** manganese monoxide, strontium nitrate, crystal surface, adsorption behavior, molecular dynamics (MD) simulation

## Abstract

Manganese monoxide (MnO), a versatile manganese oxide, is highly regarded for its potential to address heavy metal and radioactive contamination effectively. In this study, we investigated the adsorption mechanism of strontium nitrate solution on MnO crystal surfaces using molecular dynamics simulations. We examined the effects of adsorption and diffusion of ions and water molecules on three distinct MnO crystal surfaces. The results revealed significant differences in the adsorption capacities of Sr^2+^, NO_3_^−^, and H_2_O on the MnO crystal surfaces. The radial distribution function (RDF), the non-bond interaction energy (E_int_), and mean square displacement (MSD) data indicate that Sr^2+^ exhibits the strongest interaction with the MnO (111) crystal surface. This results in a shift of Sr^2+^ from outer-sphere adsorption to inner-sphere adsorption. This strong interaction is primarily due to the increase in the number and prominence of non-bridging oxygen atoms on the MnO crystal surfaces.

## 1. Introduction

Countries across the globe are actively advancing nuclear energy development due to its high energy density and low carbon emissions. The disposal of nuclear waste remains one of the foremost challenges hindering the progression of nuclear energy development [1]. Compared to various fission by-products, ^90^Sr is a major contributor to the total radioactivity in high-level liquid nuclear wastes (HLLWs) and is regarded as one of the most hazardous radioactive pollutants due to its high fission yield, long half-life, high-energy β-decay, and biotoxicity [2,3]. The efficient and rapid removal of ^90^Sr from radioactive wastewater is of critical importance. Sr^2+^ removal from radioactive wastewater is primarily achieved via precipitation [4], solvent extraction [5], and adsorption [6], with the adsorption method being particularly favored due to its simplicity and high efficiency. The effectiveness of the adsorption method is largely dependent on the selection of the adsorption material. Inorganic materials, as compared to organic materials, exhibit superior chemical and irradiation stability, rendering them more suitable for practical radioactive wastewater treatment.

Metal oxides, including Mn, Fe, and others, within inorganic materials have shown significant advancements in the removal of Sr^2+^ from radioactive wastewater. This prominence is attributed to their inorganic nature, high removal efficiencies, robust adsorption capacities, and straightforward synthesis and application procedures [7,8,9,10]. Asim et al. synthesized nano-MnO_2_ with three distinct morphologies—flower-shaped, block-shaped, and tubular—employing a microwave-assisted hydrothermal method, achieving stability in just 10 min [11]. Shen et al. prepared sodium manganese silicate via a one-pot hydrothermal approach, demonstrating an adsorption capacity for Sr^2+^ up to 249.0 mg/g [12]. Zheng et al. utilized a direct impregnation technique to deposit MnO_2_ onto HMSS, achieving a peak adsorption capacity of 138.70 mg/g [13]. Bangun et al. synthesized water-soluble coal oxide (CObt) and its composite with magnetic hematite (γ-Fe_2_O_3_) through a dual oxidation process. The results demonstrated that the materials were effective in detecting Sr^2+^ in solution under both acidic and alkaline conditions [14]. Hojae et al. identified the presence of Sr^2+^ on the hematite surface using X-ray absorption near-edge structure (XANES) and other techniques to investigate the internal ball adsorption behavior. Simultaneously, Sr^2+^ can be eluted in calcium ion solutions, leading to substitution and the formation of CaCO_3_ and CaFe_2_O_4_ [15]. Olga N. Karasyova et al. investigated the acid–base reactions and surface complexation of Sr^2+^ at the hematite/water interface through potentiometric titration at varying temperatures. Equilibrium modeling revealed that the inner-sphere complexes consisted of FeOHSr^2+^ and FeOSrOH [16]. These studies examined the adsorption properties and mechanisms of Mn and Fe oxides on Sr^2+^, with a particular focus on elucidating the form of inner-sphere complexes formed by Sr^2+^ on the hematite surface. However, it remains unclear whether Sr^2+^ on the surface of Mn oxides can form inner-sphere complexes as well. This limitation hinders the development of novel manganese oxides for Sr^2+^ removal from radioactive wastewater, thereby hindering the safe advancement of nuclear energy.

Molecular simulation serves as an invaluable tool for elucidating interactions between adsorbent materials and adsorbates, providing critical insights into the underlying adsorption mechanisms [17,18,19,20,21,22,23]. Kim et al. disclosed the adsorption behavior of Cs^+^ on todorokite via molecular dynamics (MD) simulations, noting that Cs^+^ predominantly adsorbs at the corner sites within todorokite (as an inner-sphere (IS) complex) and at edge-step sites on the external (100)/(001) surfaces (as both IS and outer-sphere (OS) complexes) [24]. Employing experimental and MD simulation techniques, Salari et al. demonstrated that increasing temperature and concentration enhances the adsorption capacity of methylene blue on MnO_2_ surfaces [25]. Guo et al., utilizing first-principles calculations, determined that a composite of MnO and graphene reduces the adsorption energy of Zn^2+^ on a microscopic scale [26]. Ilyasov et al. demonstrated via DFT simulations that the interface hydrogenation rate and spatial configuration of MnO (111) impart magnetism to single-layer graphene [27]. These studies have elucidated the interactions between the surfaces of manganese oxides, including todorokite, MnO_2_, and MnO, with Cs^+^, Zn^2+^, and other ions.

MnO, compared to other Mn oxides, adopts a Wurtzite structure within the hexagonal crystal system (space group P6_3_mc). Mn^2+^ is bonded to four equivalent O^2−^ atoms to form corner-sharing MnO_4_ tetrahedra. Active sites, such as non-bridging oxygen atoms on the crystal surface of MnO, are well defined and provide strong binding sites for Sr^2+^, in contrast to the facile hydroxylation of the MnO_2_ surface [28], which preferentially adsorbs UO^2+^ and other high-valence ions from radioactive wastewater. Furthermore, the simple hexagonal lattice of MnO enables the construction of small-scale supercells with well-established force field parameters (COMPASS force field), thereby reducing computational costs compared to manganese oxides with more complex tunneling structures, such as todorokite, which require larger models. Consequently, we selected MnO to investigate the adsorption behavior and mechanism of Sr^2+^ on the surface of manganese oxides.

This study presents, for the first time, an investigation into the adsorption behavior and mechanism of Sr^2+^ in solutions on different crystal surfaces of MnO using MD simulations. The particle density profiles (PDPs), radial distribution functions (RDFs), the network of hydrogen bonds, the non-bond interaction energies, mean square displacements, and diffusion coefficients of Sr^2+^, NO_3_^−^, and H_2_O were examined. RDF analysis revealed that Sr^2+^ formed inner-sphere complexes with non-bridging oxygen atoms on the MnO (111) crystal surface, while Sr^2+^ on other crystal surfaces formed only outer-sphere complexes. Nonbonding interactions indicated that MnO adsorbs Sr^2+^ in solution primarily through the electrostatic interaction between non-bridging oxygen atoms on the crystal surface and Sr^2+^. These microstructural and kinetic findings elucidate the adsorption behaviors and mechanisms of Sr^2+^ on the surfaces of various manganese oxide crystals, offering valuable insights into the development of high-performance manganese oxide materials for strontium removal and ensuring the safe advancement of nuclear energy.

## 2. Materials and Methods

### 2.1. Models

The modeling of interfacial adsorption of strontium nitrate solution on various MnO crystal surfaces was carried out in two stages. In the initial stage, data were retrieved from the Materials Project for MnO (mp-999539) from database version v2025.02.12 [29] post1. MnO adopts the hexagonal crystal system (space group P6_3_mc) and a lattice constant of (a = b = 3.42 Å, c = 5.35 Å, α = β = 90.00°, ɣ = 120.00°) [30]. Comprehensive details regarding the crystal structure of MnO can be found in Supplementary Materials Table S1. The XRD patterns of MnO (mp-999539) in this model are presented in Supplementary Materials Figure S1. Based on the convenience of XRD characterization and modeling calculations reported in the relevant literature, the MnO unit cell was divided into the following crystal surfaces: (100) surface (P1), (110) surface (P1), and (111) surface (P1) [30]. To facilitate the study, orthogonalization, and supercell expansion were applied to the different MnO crystal surfaces, resulting in surfaces with similar volume and area.

**Figure 1:**
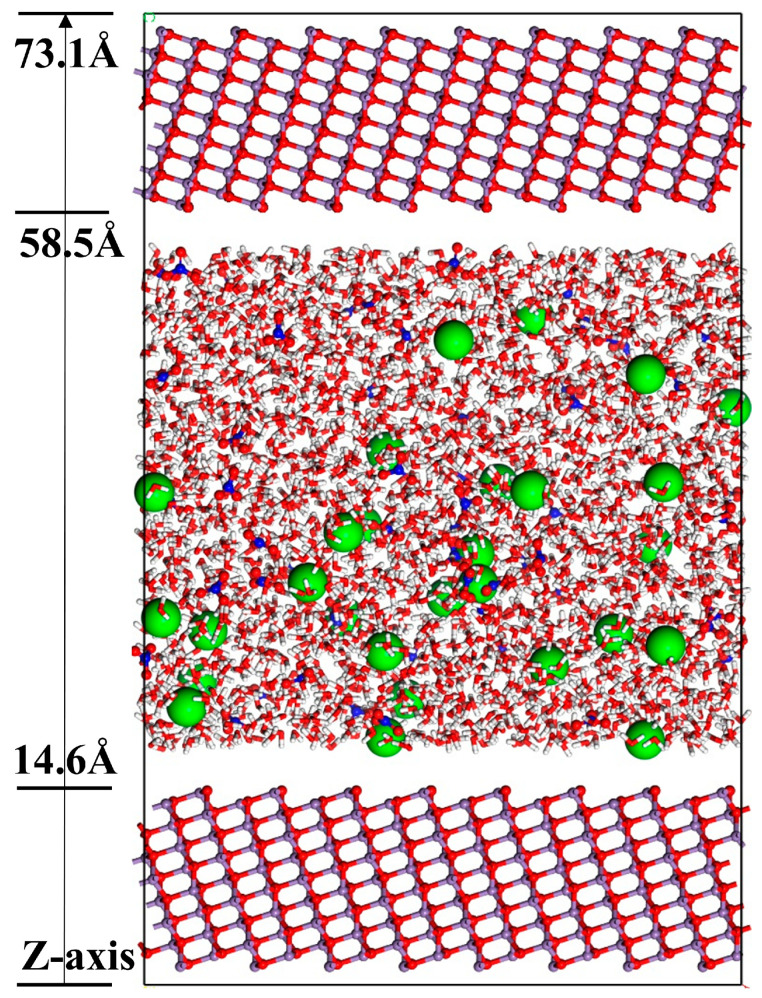
Interfacial adsorption model of strontium nitrate solution on the (111) MnO crystal surface. The solution contains 8052 atoms. Color code: white (H), red (O), blue (N), purple (Mn), and green (Sr).

In the second stage, a strontium nitrate solution model with a concentration of 0.6 mol/L was constructed, based on previous studies, containing 28 Sr^2+^ and 56 NO_3_^−^ at a density of approximately 1 g/cm^3^ [31]. To maintain symmetry and study convenience, two identical MnO crystal surfaces were positioned above and below the strontium nitrate solution. The interfacial adsorption model of strontium nitrate solution on different MnO crystal surfaces was finalized. The schematic of the interfacial adsorption model and the system information table are presented in Figure 1 and Table 1, respectively.

### 2.2. Force Field and Molecular Dynamics Simulation Details

The COMPASS (Condensed-phase Optimized Molecular Potentials for Atomistic Simulation Studies) force field offers an ideally suited potential energy function and is extensively employed in related MD simulations [32,33,34]. Consequently, this study utilized the COMPASS III force field, implementing atom-based and Ewald methods to simulate short-range van der Waals forces (with a cutoff of 12.5 Å) and long-range electrostatic forces, respectively.

The MD simulations were conducted utilizing the FORCITE module of the commercial software Materials Studio 2023. The Velocity Verlet algorithm was employed to monitor the thermodynamic properties of the system, including temperature, pressure, and total energy. The entire process was executed within the framework of the Hoover canonical ensemble (NVT) [35]. A time step of 1 fs was established, and the temperature was consistently maintained at 300 K. Temperature regulation was achieved using a Nosé thermostat, with periodic boundary conditions applied in three dimensions. Initially, the MnO crystal surfaces in the interfacial model were fixed. Subsequently, the strontium nitrate solution was allowed to diffuse freely, enabling the system to achieve equilibrium at 6000 ps within the NVT ensemble. Data analysis was carried out on the final 2000 ps of atomic trajectories to ensure robust results. Ultimately, measurements such as the density profiles, radial distribution function (RDF), network of hydrogen bonds, non-bond interaction energies, mean square displacements, and diffusion coefficients for Sr^2+^, NO_3_^−^, and H_2_O were obtained. The results of this study provide insights into the adsorption and diffusion properties and mechanisms of Sr^2+^, NO_3_^−^, and H_2_O in solutions on various MnO crystal surfaces.

### 2.3. Data Analysis

#### 2.3.1. Particle Density Profile (PDP)

The particle density profile (PDP) along the Z-axis of the simulated system was calculated to characterize the spatial distribution of ions and water molecules within different regions along the Z-axis [36]. Subsequent analyses focused on the adsorption and diffusion dynamics of particles near the MnO surface and within the solution. The formula used for the calculation is as follows.

(1) $$\rho (z)=\frac{<N(z-\frac{\Delta z}{2},z+\frac{\Delta z}{2})>}{\left(\Delta z\times S\right)}$$

Here, S refers to the surface area of the various MnO crystal surfaces, <N(z − Δz/2, z + Δz/2)> represents the number of particles between z − Δz/2 and z + Δz/2, and Δz is each bin width.

#### 2.3.2. Radial Distribution Function (RDF)

The RDF is employed to elucidate the local structure of ions in solution and at the interface [37,38]. The formula used for the calculation is as follows.

(2) $${RDF}_{A-B}(r)=\frac{1}{4\pi \rho_{B}r^{2}}\frac{dN_{A-B}}{dr}$$

RDF_A-B_ (r) describes the density of particle B at a specific distance from particle A, denoted as g(r). Here, ρ_B_ indicates the density of particle B, while dN_A−B_ represents the mean count of B particles within the interval r to r + d.

#### 2.3.3. The Network of Hydrogen Bonds

**Figure 2:**
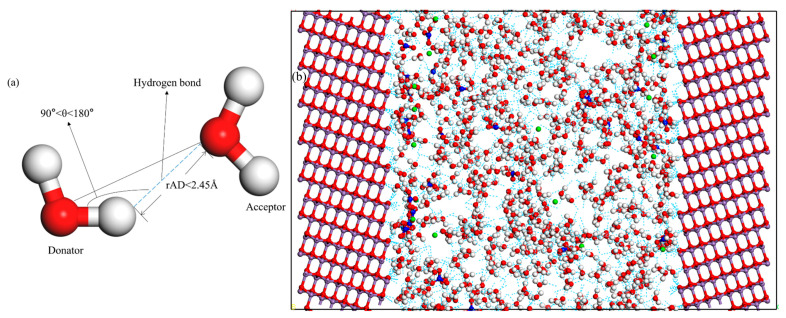
(**a**) Schematic diagram of hydrogen bond. (**b**) The network of hydrogen bonds on the MnO crystal surface. Color code: white (H), red (O), blue (N), purple (Mn), and green (Sr).

This is instrumental in delineating the local structure of water molecules and elucidating interfacial interactions. To analyze the interactions between the surface and adjacent water molecules, quantifying the number of hydrogen bonds at adsorption equilibrium is essential. As depicted in Figure 2a, the formation of hydrogen bonds requires two criteria: firstly, the distance (rAD) between adjacent hydrogen and oxygen atoms must be less than 2.45 Å; secondly, the angle formed by the Hd-Od vector and the Hd-Oa vector (where Hd is donor hydrogen, Od is donor oxygen, and Oa is acceptor oxygen) must range from 90° to 180° [39].

#### 2.3.4. The Non-Bond Interaction Energy (E_int_)

To achieve a comprehensive understanding of the thermodynamics governing the interactions between the MnO crystal surface and the strontium nitrate solution, E_int_ was computed using the equation below [36,40].

(3) $$E_{int}=E_{vdw}+E_{elec}$$

E_vdw_ and E_elec_ quantify the van der Waals and electrostatic components of the non-bond interaction energy, respectively.

#### 2.3.5. Mean Square Displacement (MSD) and Diffusion Coefficient (D)

The MSD represents the average of squared particle displacements and serves to assess the dynamic behaviors of water molecules and ions, playing a crucial role in the kinetic analysis [41]. The formula used for the calculation is as follows.

(4) $$MSD(t)=\frac{1}{n}\sum_{i=1}^{n}\langle {\left|r_{i}\left(t\right)-r_{i}(0)\right|}^{2}\rangle$$

Here, r_i_(t) and r_i_(0) denote the position and initial position, respectively, of atom i at time t, with n representing the dimensionality of the computed mean square displacement, specifically n = 3.

Furthermore, the D is determined using Einstein’s diffusion equation [42]. The formula used for the calculation is as follows.

(5) $$D=\frac{1}{6n}\underset{t\to \infty }{\lim}\frac{d}{dt}\sum_{i=1}^{n}\langle {\left|r_{i}\left(t\right)-r_{i}(0)\right|}^{2}\rangle$$

Here, t represents the MD simulation time, and the remaining variables adhere to the definitions specified in Equation (4).

## 3. Results and Discussion

### 3.1. Density Profiles of Ions and Water Molecules

**Figure 3:**
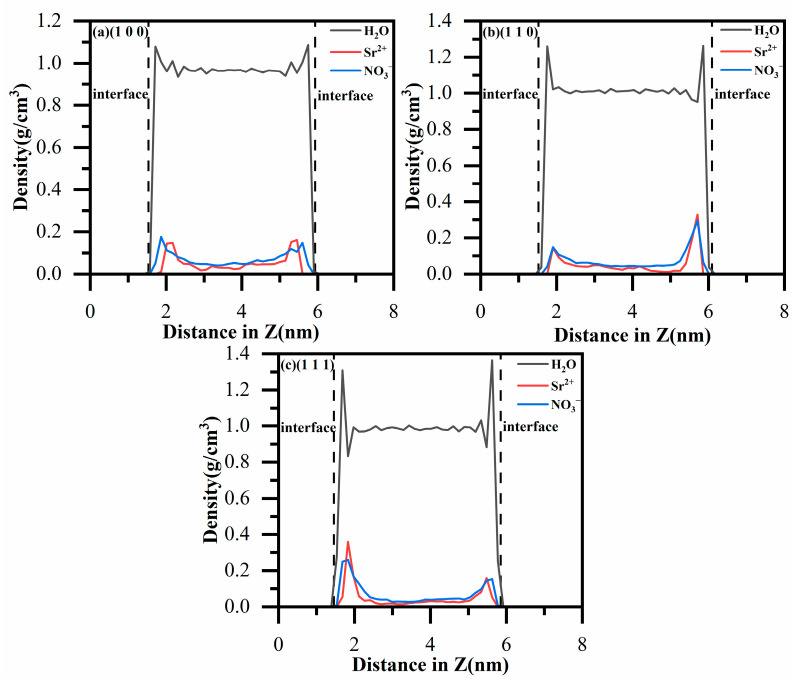
(**a**) Density profile of H_2_O, Sr^2+^, and NO_3_^−^ on the (100) crystal surface of MnO; (**b**) density profile of H_2_O, Sr^2+^, and NO_3_^−^ on the (110) crystal surface of MnO; (**c**) density profile of H_2_O, Sr^2+^, and NO_3_^−^ on the (111) crystal surface of MnO. (The black dashed lines indicate the Z-axis positions corresponding to the various MnO crystal surfaces).

Figure 3 clearly depicts the unique adsorption characteristics of H_2_O, Sr^2+^, and NO_3_^−^ on the MnO crystal surfaces. For instance, on the (100) MnO crystal surface, the high-density peaks of strontium ions occur at 21.6 Å and 54.5 Å, those of nitrate ions at 18.7 Å and 56.0 Å, and those of water molecules at 17.2 Å and 57.5 Å. Further analysis of the density distributions revealed that the high-density peaks of Sr^2+^ occurred near those of NO_3_^−^. However, the NO_3_^−^ layer is located closer to the MnO crystal surface compared to the Sr^2+^ layer. Simultaneously, the H_2_O layer is positioned even closer to the MnO crystal surface than the NO_3_^−^ layer. This finding suggests that both Sr^2+^ and NO_3_^−^ mainly adhere to the MnO surface via outer-sphere (OS) adsorption, with Sr^2+^ adsorption notably influenced by NO_3_^−^ interaction. Additionally, Figure 3 demonstrates that the highest density peak for water molecules exceeds 1 g/cm^3^. These results suggest that the density of H_2_O near the MnO crystal surface exceeds that of H_2_O in the solution. The high-density water molecule layer impedes the diffusion of both ions and water molecules. Consequently, this results in enhanced adsorption of Sr^2+^ and NO_3_^−^ on the MnO crystal surfaces [43].

**Figure 4:**
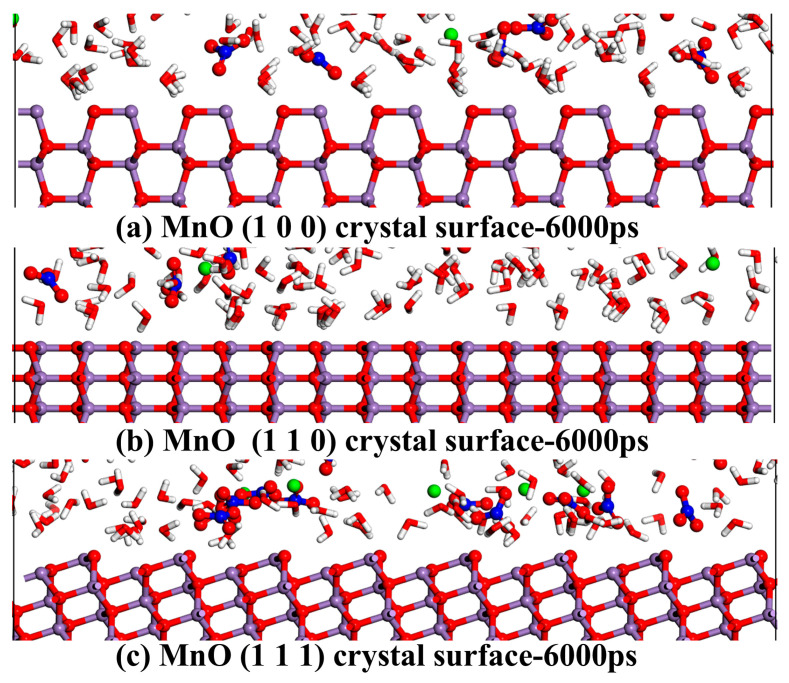
Snapshots of the interfaces within a 5 Å range on the crystal surfaces of (**a**) MnO (100) at 6000 ps, (**b**) MnO (110) at 6000 ps, and (**c**) MnO (111) at 6000 ps. Color coding: white (H), red (O), blue (N), purple (Mn), and green (Sr).

Moreover, as depicted in Figure 3, the adsorption of water molecules and ions is markedly influenced by the distinct crystal surfaces of MnO. Upon altering the crystal surface, the density of H_2_O, Sr^2+^, and NO_3_^−^ on the MnO surface generally shows an upward trend. The (100) crystal surface exhibited the highest adsorption capacity for NO_3_^−^, followed by Sr^2+^ and H_2_O, whereas the (110) and (111) crystal surfaces demonstrated the greatest adsorption for Sr^2+^, followed by NO_3_^−^ and H_2_O. Modifications to the MnO crystal surface result in an increase in the number of non-bridging oxygen atoms and the prominence of their spatial positions. Consequently, these changes increase the number of adsorption sites on the MnO crystal surfaces and enhance electronegativity, thereby improving the adsorption of water molecules and ions [44]. Figure 4 further exemplifies this phenomenon.

### 3.2. Radial Distribution Function (RDF)

**Figure 5:**
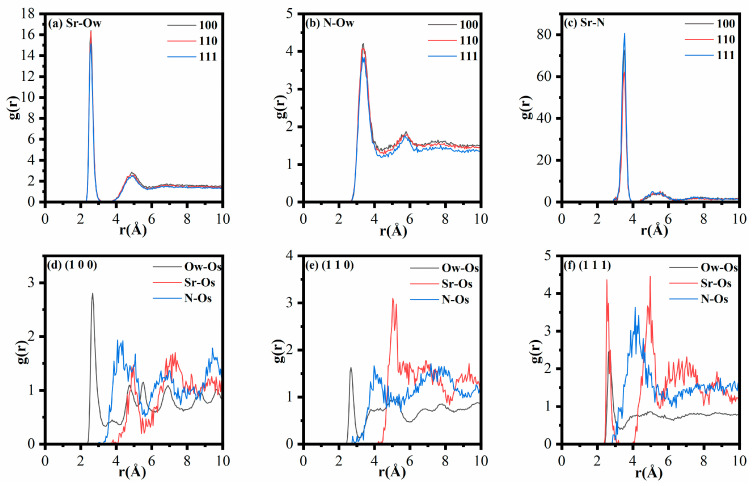
(**a**) Radial Distribution Function (RDF) of Sr-Ow on MnO crystal surfaces (100), (110), and (111); (**b**) RDF of N-Ow on MnO crystal surfaces (100), (110), and (111); (**c**) RDF of Sr-N on MnO crystal surfaces (100), (110), and (111); (**d**) RDFs of Sr-Os, N-Os, and Ow-Os on the MnO (100) crystal surface; (**e**) RDFs of Sr-Os, N-Os, and Ow-Os on the MnO (110) crystal surface; and (**f**) RDFs of Sr-Os, N-Os, and Ow-Os on the MnO (111) crystal surface. In all cases, Sr denotes Sr^2+^, N represents nitrogen atoms in NO_3_^−^, Ow refers to oxygen atoms in H_2_O, and Os refers to non-bridging oxygen atoms on the MnO crystal surface.

As depicted in Figure 5a, the Sr and Ow interaction curve displays two distinct peaks located at approximately 2.6 Å and 4.9 Å, respectively. These peaks correspond to the first and second hydration shells surrounding the central Sr^2+^. Similarly, Figure 5b illustrates that the N-Ow interaction peaks at 3.4 Å and 5.8 Å are Sr^2+^ indicative of the hydration structure of NO_3_^−^. The first hydration shells of Sr-Ow and N-Ow basically align with previous scholarly reports, as shown in Figure 5a,b (Sr-Ow: 2.6–2.7 Å; N-Ow: 3.5 Å) [31,45,46]. It is notable that the distance between the two peaks of Sr-Ow is less than that between the two peaks of N-Ow. The findings demonstrate that the clusters of Sr^2+^ and H_2_O are denser than those of NO_3_^−^ and H_2_O. Furthermore, as evidenced in Figure 5a,b, the alteration of the MnO crystal surfaces does not markedly affect the radial distribution function (RDF) of Sr-Ow and N-Ow. This suggests that the modification of the MnO crystal surfaces exerts minimal influence on the hydration dynamics of Sr^2+^ and NO_3_^−^ within the solution. As shown in Figure 5c, the Sr-N RDF curves on different MnO crystal surfaces exhibit subtle changes, with two prominent peaks: a strong peak at 3.5 Å and weaker peaks at 5.3 Å, 5.2 Å, and 5.1 Å, respectively. The results confirm that changes in the MnO crystal surfaces do not affect the coordination mechanism of Sr^2+^ and NO_3_^−^ on these surfaces. In an aqueous solution, Sr^2+^ typically adopts an octahedral configuration with eight water molecules (Sr^2+^ (H_2_O)_8_) [47], while NO_3_^−^ can form clusters by partially substituting water molecules, such as Sr^2+^-(H_2_O)_n_-(NO_3_^−^)_8−n_ [48,49]. The prominent peaks indicate stable bond lengths between Sr^2+^ and NO_3_^−^, governed by direct electrostatic forces, while the less pronounced peaks correspond to the Sr-Ow-N bond lengths, which arise from indirect ionic interactions [41]. The prominent peaks of Sr-N bonds on the (111) MnO crystal surface are considerably higher than those on other crystal surfaces. Additionally, the less prominent peak of the Sr-Ow-N bond on the (111) MnO crystal surface is shifted closer to the left side of the transverse axis compared to the peaks on other MnO crystal surfaces. These results suggest that the direct electrostatic and indirect ionic interactions between Sr^2+^ and NO_3_^−^ are most pronounced, with the bonding being the tightest and most stable on the (111) MnO crystal surface.

**Figure 6:**
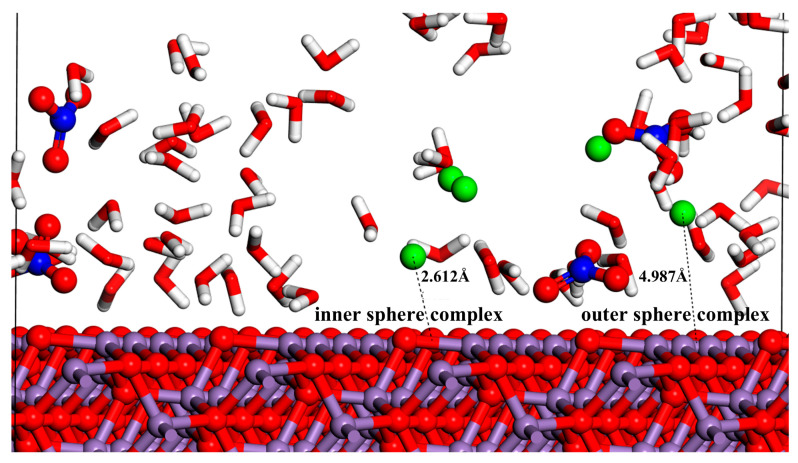
Snapshots of Sr^2+^ inner-sphere (IS) adsorption and outer-sphere (OS) adsorption at the MnO (111) crystal surface at 6000 ps. Color code: white (H), red (O), blue (N), purple (Mn), and green (Sr).

Figure 5a–c demonstrate that the RDFs of Sr-Os, N-Os, and Ow-Os feature pronounced peaks across various crystal surfaces. The MnO crystal surface displays significant adsorption capacities for Sr^2+^, NO_3_^−^, and H_2_O. Transitioning from the (100) to the (110) and (111) crystal surface, the Sr-Os curve shows an upward trajectory, whereas the N-Os and Ow-Os curves first decline before demonstrating an upward trend. These results suggest that the adsorption of hydrated Sr^2+^ on the MnO crystal surface increases with the number of non-bridging oxygen atoms and their more pronounced localization on the surface. Specifically, as shown in Figure 5d, the strong peak positions of Sr-Os on the (100) crystal surface occur at 4.9 Å and 6.8 Å, with their corresponding g(r) values both smaller than that of Ow-Os. For the (110) crystal surface, as shown in Figure 5e, the strong peak positions of Sr-O and Sr-Os are at 5.0 Å and 6.9 Å, with their corresponding g(r) values both larger than those of Ow-Os. On the (111) crystal surface, as shown in Figure 5f, the strong peak positions of Sr-O and Sr-Os occur at 2.5 Å and 5.0 Å, with their corresponding g(r) values both larger than those of the Ow-Os counterpart. The position of the Sr-Ow hydration shell is typically 2.6 Å [50]. Therefore, Sr^2+^ exhibits only outer-sphere adsorption behavior on the MnO (100) and (110) crystal surfaces, while both inner-sphere and outer-sphere adsorption behaviors are present on the (111) crystal surface, as illustrated in Figure 5d–f. This conclusion is further corroborated by the data presented in Figure 6. The Sr^2+^ ions form Sr-Os bonds with non-bridging oxygen atoms on the (111) crystal surface of MnO, as indicated by the coordination numbers in Figure S2 of the Supplementary Materials. Conversely, the adsorption capacity for hydrated NO_3_^−^ initially diminishes and then subsequently increases. Additionally, the RDF curve shown in Figure 5d reveals that the peak for N-Os on the (100) crystal surface surpasses that for Sr-Os. Unlike the (110) and (111) crystal surfaces, the (100) crystal surface demonstrates a higher adsorption capacity for hydrated NO_3_^−^ compared to hydrated Sr^2+^. Even though the (100) crystal surface has fewer non-bridging oxygen atoms compared to other crystal surfaces, these atoms are more isolated and sparsely distributed. Consequently, as shown in Figure 5d,e, this leads to the N-Os peak on the (100) crystal surface being higher than on the (110) crystal surface. Further analysis revealed that changes in the crystal surface significantly influenced the adsorption of Sr^2+^ on the MnO crystal surface, followed by NO_3_^−^ and H_2_O.

In summary, the adsorption mechanisms of Sr^2+^ and NO_3_^−^ on MnO crystal surfaces were elucidated. On the one hand, with changes in the MnO crystal surfaces, the number of non-bridging oxygen atoms increases, and their spatial positions become more pronounced, resulting in more adsorption sites and enhanced electronegativity. Consequently, this results in the accumulation and partial desolvation of hydrated Sr^2+^ ions on the (111) MnO crystal surface, which subsequently attracts NO_3_^−^ and its hydrated form through electrostatic forces, forming clusters and complexes. Ultimately, this process culminates in the adsorption of NO_3_^−^ on the MnO crystal surfaces. Conversely, while the electronegativity of the MnO crystal surfaces enhances the repulsive forces between NO_3_^−^ and these surfaces, it simultaneously increases the adsorption capacity of Sr^2+^-(H_2_O)_n_-(NO_3_^−^)_8−n_ complexes. The total adsorption of NO_3_^−^ onto MnO crystal surfaces surpasses the repulsive forces. Therefore, NO_3_^−^ continues to adsorb near the MnO crystal surfaces. Supporting evidence from other studies supports this conclusion. For example, Wang et al. showed that enhancing the electronegativity of the geopolymer surface improves the ion adsorption capacity [21,51]. Similarly, Xu et al. observed that an increase in the number of exposed adsorption active sites on α-Fe_2_O_3_ nanocrystals significantly improved the ion adsorption capacity [52].

### 3.3. The Network of Hydrogen Bonds

Figure 2a depicts the conditions necessary for hydrogen bond formation. Figure 2b illustrates two distinct categories of hydrogen bonds: those present in solutions, primarily Ow-Ow and N-Ow, and those at the interface, predominantly Ow-Os and N-Os. As demonstrated in Table 2, at the outset of the simulation, the interface initially contained no hydrogen bonds. This indicates that before the onset of adsorption, the interface is devoid of hydrogen bonds. As the MD simulation progresses, ions and water molecules accumulate at the interface, forming a dense solution layer. Upon reaching the adsorption equilibrium at 6000 ps, the number of hydrogen bonds at various interfaces stabilizes at 322, 204, and 241, respectively. This results in an increased number of hydrogen bonds at the interface. Once a stable adsorption state is achieved on the MnO surface, the number of hydrogen bonds at the interface remains relatively constant. Additionally, as illustrated in Table 1 and Figure 4, the number and spatial positioning of non-bridging oxygen atoms on various MnO crystal surfaces influence the number of hydrogen bonds at the interface [44]. Despite fewer non-bridging oxygen atoms on the (100) crystal surface compared to other crystal surfaces, these atoms are more isolated and sparsely distributed on their surface. Relative to other crystal surfaces, the water molecules at the interface of the (100) surface are arranged more orderly, leading to a higher formation of hydrogen bonds [53]. The (111) and (110) crystal surfaces possess an identical number of non-bridging oxygen atoms on their surfaces. However, the non-bridging oxygen atoms on the (111) crystal surface are more pronounced. Consequently, this leads to a greater number of hydrogen bonds at the interface of the (111) crystal surface compared to the (110) surface. Thus, this clarifies the differences in the distribution and number of hydrogen bonds on the MnO crystal surfaces. This can be attributed to variations in the number and spatial arrangement of non-bridging oxygen atoms on the crystal surfaces.

### 3.4. The Non-Bond Interaction Energy

Table 3 shows that the non-bond interaction energy, including its components—electrostatic and van der Waals energies—are uniformly negative. The adsorption process constitutes a spontaneous exothermic reaction. Additionally, the van der Waals energy is about an order of magnitude lower than the electrostatic energy. Electrostatic attraction serves as the primary driving force in this adsorption process. Given the similarity in the areas of the three crystal surfaces and the significant differences in their adsorption energies, direct comparisons of the non-bond interaction energy data across different surfaces are feasible [54]. For the (100) crystal surface, the absolute non-bond interaction energy value is the highest. This can be attributed to the highest adsorption capacity for water molecules at the interface. This aligns with the data in Figure 4 and Figure 5d, showing that the Ow-Os on the (100) crystal surfaces have the most pronounced radial distribution function (RDF) peaks and the highest number of hydrogen bonds. Considering that the (100) crystal surface has fewer non-bridging oxygen atoms than other surfaces, it follows that its adsorption capacity for Sr^2+^ is limited. The absolute non-bond interaction energy values for the (111) and (110) crystal surfaces rank second and third, respectively. Notably, the number of non-bridging oxygen atoms on their surfaces is identical and exceeds that on the (100) crystal surfaces, as seen in Table 1. Consequently, their adsorption effectiveness for Sr^2+^ surpasses that of the (100) crystal surfaces. This corresponds with the findings in Figure 5d–f, which reveal that the RDF curve peaks for Sr-Os on the (111) and (110) crystal surfaces are higher than those on the (100) surface. Concurrently, the spatial positioning of the non-bridging oxygen atoms on the (111) crystal surface is more pronounced compared to that on the (110) crystal surface. Consequently, this leads to more effective adsorption of Sr^2+^, NO_3_^−^, and H_2_O on the (111) crystal surface compared to the (110) surface. This is corroborated by the findings in Figure 5e,f, which show that the RDF curve peaks for Sr-Os, N-Os, and Ow-Os on the (111) crystal surface exceed those on the (110) surface. Additionally, the non-bond interaction energy and network of hydrogen bonds can be used to infer the enthalpy change of MnO adsorption from a strontium nitrate solution at varying temperatures. The enthalpy associated with the interaction between the MnO crystal surface and the strontium nitrate solution decreases as the temperature increases. For further details, refer to the Supplementary Materials.

### 3.5. Mean Square Displacement (MSD) and Diffusion Coefficient (D)

**Figure 7:**
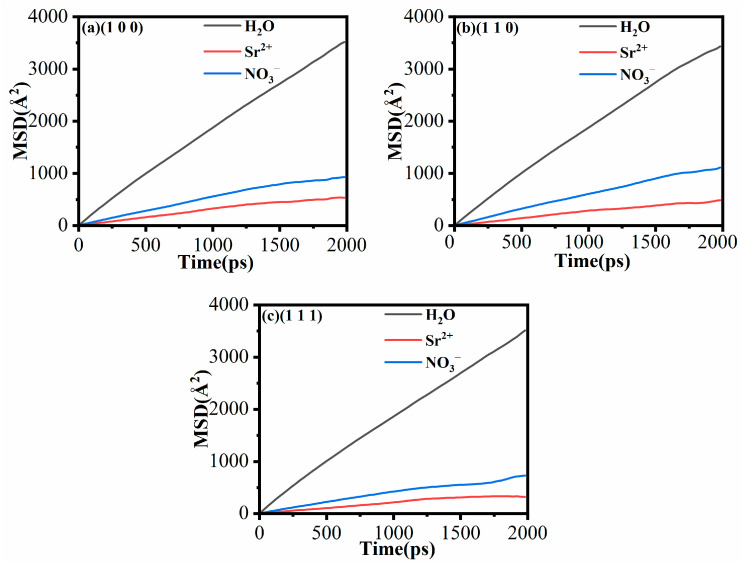
(**a**) Mean square displacement (MSD) of H_2_O, Sr^2+^, and NO_3_^−^ on the (100) MnO crystal surface; (**b**) MSD of H_2_O, Sr^2+^, and NO_3_^−^ on the (110) MnO crystal surface; (**c**) MSD of H_2_O, Sr^2+^, and NO_3_^−^ on the (111) MnO crystal surface.

Figure 7 illustrates the kinetics of Sr^2+^, NO_3_^−^, and H_2_O on different MnO crystal surfaces. As shown in Figure 7a, H_2_O exhibits the highest diffusion rate on the (100) MnO crystal surface, followed by NO_3_^−^ and, lastly, Sr^2+^. This is primarily due to the high adsorption capacity of the MnO crystal surface for Sr^2+^, which effectively limits the diffusion of Sr^2+^. As shown in Figure 7b, the diffusion of Sr^2+^ and H_2_O decreases further as the transition occurs from the (100) to the (110) crystal surface, while the diffusion of NO_3_^−^ slightly increases. On the (111) crystal surface, the mobility of both Sr^2+^ and NO_3_^−^ decreased significantly. As shown in Figure 7b,c, the mean square displacements (MSDs) of ions and water molecules on the (110) and (111) MnO crystal surfaces indicate that the mobility of H_2_O significantly exceeds that of Sr^2+^ and NO_3_^−^. This phenomenon can be attributed to three primary factors. One factor is the relatively weak interactions between water molecules, primarily governed by hydrogen bonds, which facilitate rapid diffusion in solutions. The second factor involves non-bridging oxygen atoms on the MnO crystal surface, whose electronegativity attracts Sr^2+^, with diffusion subsequently limited by strong electrostatic forces. The final factor is the formation of dense clusters of hydrated Sr^2+^ and hydrated NO_3_^−^, coupled with the complexation of hydrated Sr^2+^, hydrated NO_3_^−^, and H_2_O on the MnO crystal surface [41].

As shown in Table 4, the diffusion coefficients of H_2_O, Sr^2+^, and NO_3_^−^ derived from the MD simulations are consistent with previously reported results [31,51]. Table 4 shows that the diffusivity of H_2_O, NO_3_^−^, and Sr^2+^ decreases sequentially on the same MnO crystal surface. Additionally, Table 1 and Figure 4 show that changes in the crystal surface result in an increase in the number of non-bridging oxygen atoms and a more pronounced spatial localization on the MnO crystal surface. This results in a significant decrease in the diffusion coefficients of Sr^2+^ and NO_3_^−^, as shown in Table 4. Therefore, it can be inferred that the increase in the number and prominence of non-bridging oxygen atoms on the MnO crystal surface enhances its adsorption capacity for Sr^2+^ and NO_3_^−^. This limitation on the diffusion of ions and water molecules aligns with the enhanced adsorption capacity on the MnO crystal surface.

## 4. Conclusions

In this study, comprehensive molecular dynamics (MD) simulations were employed to investigate the adsorption behavior and underlying mechanisms of strontium nitrate solution on the crystal surfaces of MnO. First, we observed that Sr^2+^ exhibits distinct adsorption behaviors and mechanisms on the various crystal surfaces of MnO. Sr^2+^ can form inner-sphere complexes with non-bridging oxygen atoms on the (111) crystal surface of MnO, whereas on the (100) and (110) crystal surfaces, it forms only outer-sphere complexes. Secondly, this phenomenon arises from the variation in the number and spatial arrangement of non-bridging oxygen atoms across the crystal surfaces of MnO. This variation governs the difference in adsorption sites and electronegativity, which, in turn, influences the adsorption of Sr^2+^, NO_3_^−^, and H_2_O. The (111) crystal surface has the highest concentration and most prominent spatial arrangement of non-bridging oxygen atoms, leading to its strongest adsorption of Sr^2+^. The (100) crystal surface contains the fewest non-bridging oxygen atoms, resulting in the weakest adsorption of Sr^2+^. The (110) crystal faces exhibit intermediate properties. Furthermore, the dominant forces responsible for the adsorption of Sr^2+^, NO_3_^−^, and H_2_O on the MnO crystal surfaces differ. Sr^2+^ and NO_3_^−^, along with their constituent cluster complexes, are adsorbed primarily through electrostatic interactions, while H_2_O is adsorbed through hydrogen bonds. Finally, the present study offers valuable insights into the development of high-efficiency manganese oxides for strontium removal, thereby contributing to the safe advancement of nuclear energy.

## Figures and Tables

**Table 1:** Details of the particulate systems studied in this work *.

Model	a/Å	b/Å	c/Å	α/(deg)	β/(deg)	γ/(deg)	$N_{MnO}$	$N_{H_{2}O}$	$N_{{Sr}^{2+}}$	$N_{{NO}_{3}^{-}}$	$N_{S-NBO}$
(100)	44.46	42.78	74.65	90.00	90.00	90.00	2080	2600	28	56	208
(110)	42.78	41.47	76.11	90.00	90.00	90.00	2016	2600	28	56	224
(111)	44.91	41.47	73.06	90.00	90.00	90.00	1904	2600	28	56	224

* Footnote to table: N represents the number of particles. N_S-NBO_ represents the number of non-bridging oxygen atoms on the MnO crystal surface.

**Table 2:** The number of hydrogen bonds formed by strontium nitrate solution on MnO crystal surfaces.

Interface	(100)	(110)	(111)
The number of hydrogen bonds (0 ps)	0	0	0
The number of hydrogen bonds (6000 ps)	322	204	241

**Table 3:** The non-bond interaction energies between strontium nitrate solutions and different MnO crystal surfaces.

MnO	(100)	(110)	(111)
E_int_ (kcal/mol)	−4257.04	−2474.91	−3181.38
E_elec_ (kcal/mol)	−4112.27	−1729.95	−2521.50
E_vdw_ (kcal/mol)	−144.77	−744.96	−659.88

**Table 4:** Diffusion coefficients (Ds) of H_2_O, Sr^2+^, and NO_3_^−^ on different MnO crystal surfaces: (100), (110), and (111).

MnO	(100)	(110)	(111)
H_2_O (1 × 10^−5^ cm^2^/s)	2.77	2.70	2.78
Sr^2+^ (1 × 10^−5^ cm^2^/s)	0.34	0.33	0.18
NO_3_^−^ (1 × 10^−5^ cm^2^/s)	0.61	0.88	0.46

## Data Availability

The original contributions presented in this study are included in the article. Further inquiries can be directed to the corresponding author.

## Supplementary Materials

The following supporting information can be downloaded at: https://www.mdpi.com/article/10.3390/ma18081752/s1, Figure S1: The XRD of MnO (mp-999539); Table S1: MnO crystal structures are available in the Materials Project database; Figure S2: The coordination number (CN) of Sr-Os on various crystal surfaces of MnO; effect of temperature change and enthalpy of the adsorption process.
