# Supplementary material for: Understanding the Adsorption Behavior of Different Crystal Surfaces of Manganese Monoxide to Strontium Nitrate Solutions: A Molecular Dynamics Simulation
Source: Materials (Basel). 2025 Apr 11;18(8):1752. doi: 10.3390/ma18081752 (PMC12028766; doi:10.3390/ma18081752)
Supplement: Supplementary file 1 [file materials-18-01752-s001.zip › materials-3510289-supplementary.pdf]

Supplementary Materials

S1. The XRD of MnO (mp-999539)

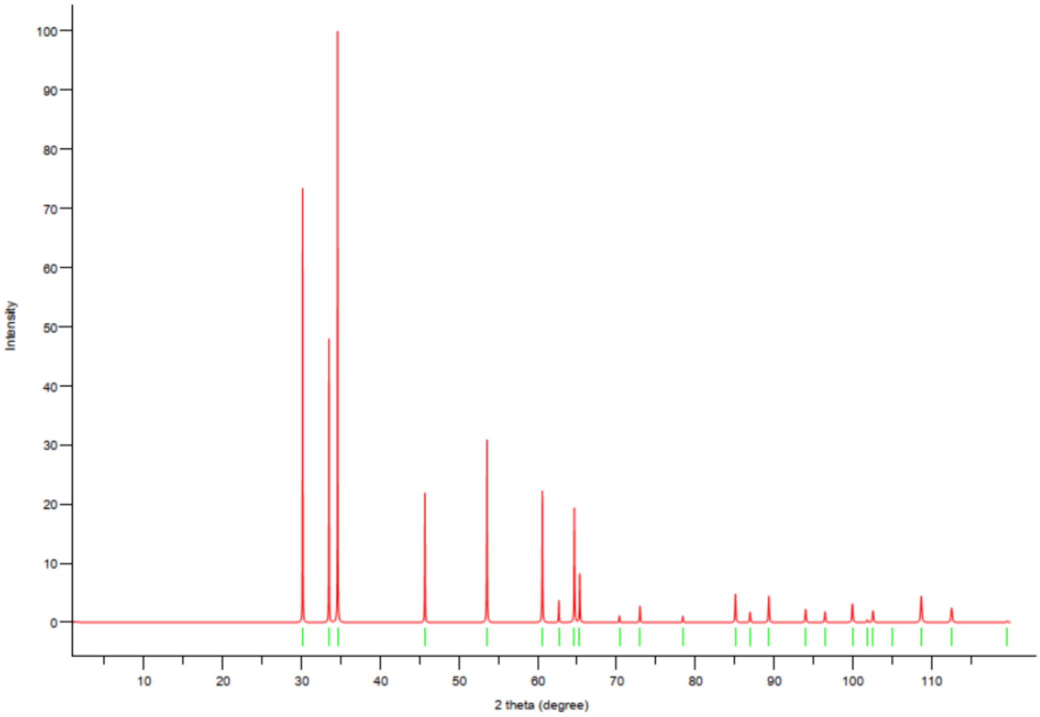

Figure S1. The XRD of MnO (mp-999539).

Data retrieved from the Materials Project for MnO (mp-999539) from database version v2025.02.12. post1.

S2. MnO crystal structures are available in the Materials Project database.

Table S1. MnO crystal structures are available in the Materials Project database.

Experimentally observed	Material ID	Formula	Crystal System	Space Group Symbol	Sites
No	mp-1232659	MnO	Orthorhombic	Pmmn	8
Yes	mp-19006	MnO	Cubic	$Fm\bar{3}m$	4
No	mp-2831286	MnO	Orthorhombic	Cmc2_1	8
Yes	mp-999539	MnO	Hexagonal	$P6_3mc$	4
No	mp-1238899	MnO	Cubic	$F\bar{4}3m$	2
No	mp-1238773	MnO	Hexagonal	$P\bar{6}m2$	2

Data retrieved from the Materials Project for MnO from database version v2025.02.12. post1.

S3. The coordination number (CN) of Sr-Os

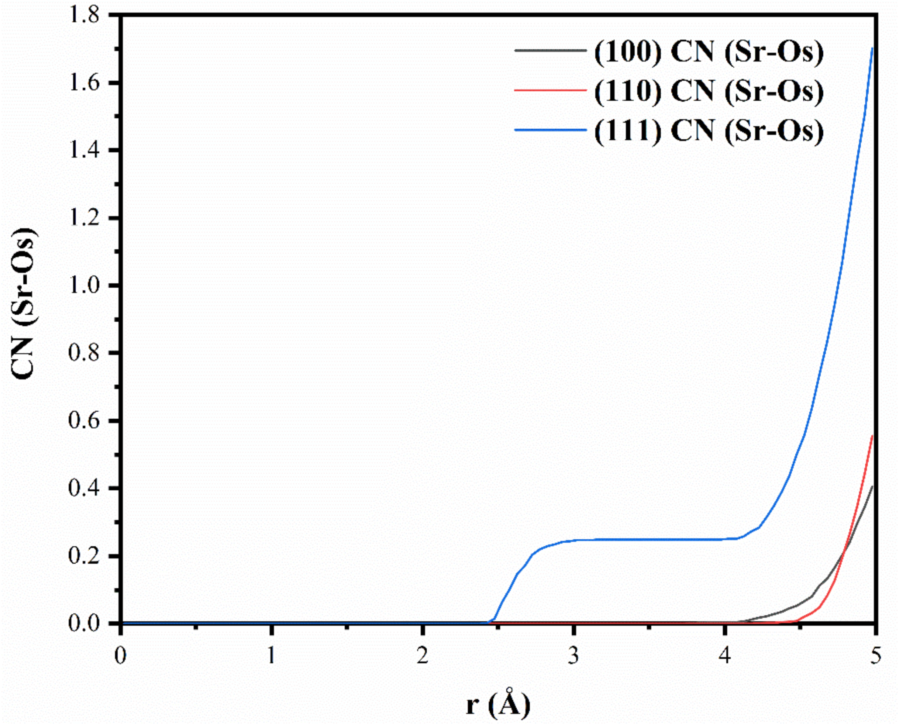

Figure S2. The coordination number (CN) of Sr-Os on various crystal surfaces of MnO. The formula is as follows:

$$CN(r) = \sum_{r=0}^{r} 4\pi r^2 \rho_B g(r) dr \qquad (S1)$$

$RDF_{A\text{-}B}$ (r) describes the density of particle B at a specific distance from particle A, denoted as g(r). Here, ϱ_B indicates the density of particle B.

S4. Effect of temperature change and enthalpy of the adsorption process

At solid-liquid interfaces, the non-bond interaction energy tends to be related to the Gibbs free energy. Consequently, we employ Equations (S2) and (S3) to derive Equation (S4).

$$\Delta G = \Delta H - T\Delta S \qquad (S2)$$

$$Eint \approx \Delta G \qquad (S3)$$

$$\Delta H = Eint + T\Delta S \qquad (S4)$$

Equation (S2) represents the Gibbs free energy formula. Equation (S3) represents approximating the non-bond interaction energy between the MnO crystal surface and strontium nitrate solution to the Gibbs free energy. Equation (S4) represents the equation for the enthalpy change between the MnO crystal surface and the strontium nitrate solution.

ΔG represents the Gibbs free energy change; ΔH denotes the enthalpy change; T refers to the absolute temperature; ΔS corresponds to the entropy change; and Eint represents the non-bond interaction energy.

Electrostatic interactions dominate the non-bond interaction energies between the three MnO crystal faces and the strontium nitrate solution. Additionally, the network of hydrogen bonds forms between the three MnO crystal surfaces and the strontium nitrate solution. As temperature increases, the electrostatic interaction energy at the solid-liquid interface

weakens, followed by a reduction in the non-bond interaction energy. Simultaneously, as the temperature rises, the network of hydrogen bonds weakens, increasing entropy change (ΔS). The effect of non-bond interaction energy outweighs that of entropy increase, resulting in a decrease in the overall enthalpy change (ΔH) between the MnO crystal surface and the strontium nitrate solution as temperature increases.
